# Supplementary figures and images for: Do chimpanzees (Pan troglodytes) mentally represent collaboration?: Action-learning and communication in a partnered task
Source: PLoS One. 2025 Jun 6;20(6):e0325418. doi: 10.1371/journal.pone.0325418 (PMC12143569; doi:10.1371/journal.pone.0325418)

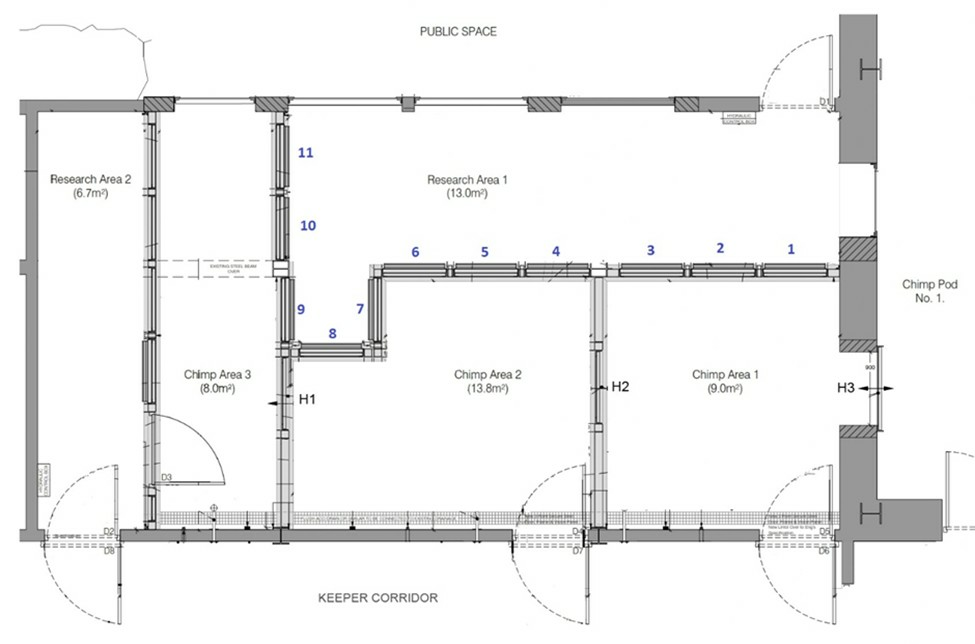

Supplement: S1 Figure — “Research Area” indicates areas where human researchers can safely sit or stand to conduct experimental research with the chimpanzees at Edinburgh Zoo. All research areas are separated from chimp areas by metal walls or by mesh/polycarbonate panels (numbered 1–11 below). “Chimp Area” indicates areas where chimpanzees can enter or exit freely during research times. These areas are accessed via one of the chimpanzee’s indoor enclosure pods (Chimp Pod No. 1), and are divided by hydraulic doors (H1, H2, and H3), which are always open during research times. This experiment was conducted in the alcove contained by windows 7, 8, and 9. (TIF) [file pone.0325418.s001.tif]

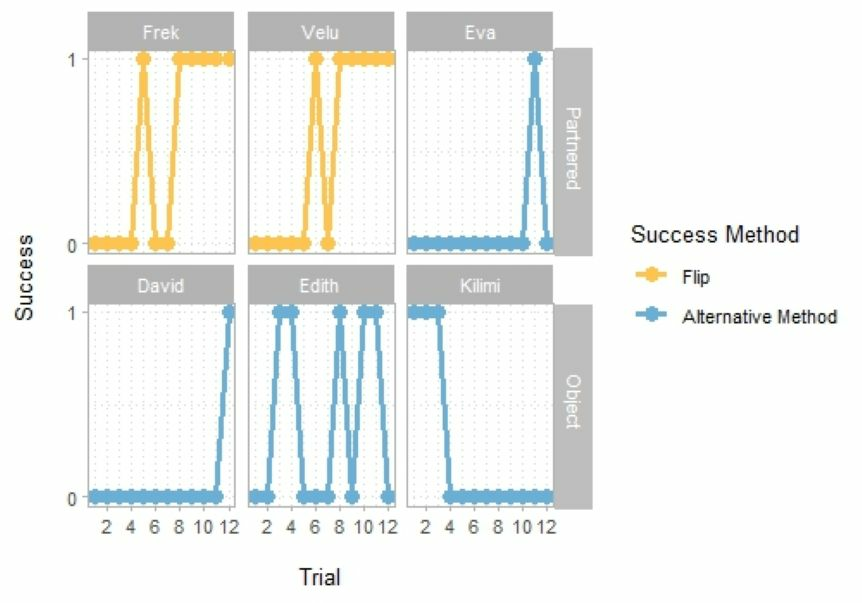

Supplement: S2 Figure — Subjects in the partnered learning condition, specifically those who used the “flip” method of success, tended to replicate their success consistently over subsequent trials, while those in the object condition, using alternative methods, succeeded only sporadically across trials. (TIF) [file pone.0325418.s002.tif]
