# Supplementary material for: Do chimpanzees (Pan troglodytes) mentally represent collaboration?: Action-learning and communication in a partnered task
Source: PLoS One. 2025 Jun 6;20(6):e0325418. doi: 10.1371/journal.pone.0325418 (PMC12143569; doi:10.1371/journal.pone.0325418)
Supplement: S1 Table — All subjects received one initial session of pre-test 2 before attempting to meet passage criteria, thus the minimum possible number of sessions is 3. (DOCX) [file pone.0325418.s003.docx]

| **Subject** | **Number of Sessions in Pre-Test 2** |
| --- | --- |
| **Eva** | 3 |
| **Edith** | 3 |
| **Louis** | 3 |
| **David** | 5 |
| **Kilimi** | 5 |
| **Lucy** | 7 |
| **Liberius** | 7 |
| **Qafzeh** | 7 |
| **Velu** | 7 |
| **Frek** | 11 |
