# Supplementary material for: Do chimpanzees (Pan troglodytes) mentally represent collaboration?: Action-learning and communication in a partnered task
Source: PLoS One. 2025 Jun 6;20(6):e0325418. doi: 10.1371/journal.pone.0325418 (PMC12143569; doi:10.1371/journal.pone.0325418)
Supplement: S2 Table — (DOCX) [file pone.0325418.s004.docx]

| **Subject** | **Number of Repeated Trials** |
| --- | --- |
| **Eva** | 1 |
| **Edith** | 6 |
| **Louis** | 1 |
| **David** | 4 |
| **Kilimi** | 6 |
| **Lucy** | 0 |
| **Liberius** | 4 |
| **Qafzeh** | 0 |
| **Velu** | 0 |
| **Frek** | 1 |
