# Supplementary material for: Do chimpanzees (Pan troglodytes) mentally represent collaboration?: Action-learning and communication in a partnered task
Source: PLoS One. 2025 Jun 6;20(6):e0325418. doi: 10.1371/journal.pone.0325418 (PMC12143569; doi:10.1371/journal.pone.0325418)
Supplement: S3 Table — The phase or trials listed for each subject in each time period indicate the starting (S) and final (F) piece of testing completed within that time period. Any phase that was incomplete and restarted in the next period following a delay is indicated with an (x). (DOCX) [file pone.0325418.s005.docx]

| **Subject** |  | **Sep 2019 – Feb 2020** | **Mar – Sep 2020** | **Oct – Dec 2020** | **Jan – Apr 2021** | **May 2021** | **June – Sep 2021** | **Oct – Nov 2021** |
| --- | --- | --- | --- | --- | --- | --- | --- | --- |
| **Velu** | S | pre-test 1 | ***COVID-19 lockdown*** | 10 refresh trials | ***COVID-19 lockdown*** | n/a | ***research delays due to COVID-19 lockdowns*** | n/a |
|  | F | test phase |  | completed study |  | n/a |  | n/a |
| **Eva** | S | pre-test 1 |  | 10 refresh trials |  | n/a |  | n/a |
|  | F | 100 learning trials |  | completed study |  | n/a |  | n/a |
| **Kilimi** | S | pre-test 1 |  | 30 learning trials |  | n/a |  | n/a |
|  | F | 70 learning trials |  | completed study |  | n/a |  | n/a |
| **Louis** | S | pre-test 1 |  | 33 learning trials |  | n/a |  | n/a |
|  | F | 67 learning trials |  | completed study |  | n/a |  | n/a |
| **Lucy** | S | pre-test 1 |  | 10 refresh trials |  | 10 refresh trials |  | n/a |
|  | F | test phase (x) |  | test phase |  | completed study |  | n/a |
| **Edith** | S | pre-test 1 |  | 55 learning trials |  | 10 refresh trials |  | n/a |
|  | F | 45 learning trials |  | test phase (x) |  | completed study |  | n/a |
| **Frek** | S | pre-test 1 |  | block 8, pre-test 2 |  | 67 learning trials |  | n/a |
|  | F | 7 blocks pre-test 2 |  | 33 learning trials |  | completed study |  | n/a |
| **Qafzeh** | S | pre-test 1 |  | 68 learning trials |  | 10 learning trials |  | 10 refresh trials |
|  | F | 32 learning trials |  | 97 learning trials |  | test phase |  | completed study |
| **David** | S | pre-test 1 |  | 74 learning trials |  | 27 learning trials |  | 10 refresh trials |
|  | F | 26 learning trials |  | 73 learning trials |  | test phase |  | completed study |
| **Liberius** | S | pre-test 1 |  | 55 learning trials |  | 10 refresh trials |  | 10 refresh trials |
|  | F | 45 learning trials |  | test phase (x) |  | test phase (x) |  | completed study |
