# Supplementary material for: Do chimpanzees (Pan troglodytes) mentally represent collaboration?: Action-learning and communication in a partnered task
Source: PLoS One. 2025 Jun 6;20(6):e0325418. doi: 10.1371/journal.pone.0325418 (PMC12143569; doi:10.1371/journal.pone.0325418)
Supplement: S4 Table — (DOCX) [file pone.0325418.s006.docx]

| **Behaviour** | **Definition** |
| --- | --- |
| **tap** | the subject taps or raps their fingers against the table, window, or apparatus from any side, including beneath. Taps may be single or repetitive and should be counted only once if the subject has not removed their fingers from the mesh between taps. Note that a tap or contact between the fingers and the apparatus performed as part of an action to manipulate the apparatus (i.e., pull the paper, peel the blocks from the table, touch the see-saw, or fish for the grape) should not be counted. Note that taps from beneath, which may lift or rattle the table, should be counted. |
| **finger thrust** | the subject thrusts the fingers (or single finger) of one hand through the mesh. Thrusts may be single or repetitive and should be counted only once unless the subject completely ceases the action (either by removing the fingers entirely or by leaving the fingers through the mesh passively) and then subsequently begins again. |
| **head nod** | the subject moves their head up and down (nodding) or side to side (shaking, i.e., ear moves toward shoulder) repeatedly (at least two movements) with the eyeline directed at E. |
| **pass paper** | the subject pushes a strip of paper (either having already pulled the current piece or using one from the floor) back through the mesh either partially or completely. All motions/thrusts/taps associated with this activity should be counted as one event, unless they completely cease the behaviour (1 second elapses between efforts or the subject engages in another behaviour and then returns to this behaviour) and then resume while the paper is still partially in the mesh. |
| **raspberry** | the subject produces a lip buzzing/raspberry sound/mouth gesture. This sound/gesture may be single or repeated but should only be counted twice if the behaviour fully ceases before beginning again. |
| **grumble** | the subject produces an audible, low-pitched vocalization, akin to a grunt or grumble |
| **whine** | the subject produces a high-pitched vocalization akin to a whine or cry (not a scream or call) |
| **hand fling** | the subject raises their hand upward, with the palm facing away from E, and produces a flinging gesture toward the shoulder |
| **present mouth** | the subject presents their lips/mouth, open or closed, such that at least one lip protrudes through the mesh. Note that the use of the lips, tongue, or teeth in effort to pull the paper or manipulate the apparatus (e.g., the blocks) should not be counted. |
