# Supplementary material for: Do chimpanzees (Pan troglodytes) mentally represent collaboration?: Action-learning and communication in a partnered task
Source: PLoS One. 2025 Jun 6;20(6):e0325418. doi: 10.1371/journal.pone.0325418 (PMC12143569; doi:10.1371/journal.pone.0325418)
Supplement: Attachment — Submitted filename: PONE-D-25-05158_ReviewerComments.pdf [file pone.0325418.s007.pdf]

Title: **Do chimpanzees (Pan troglodytes) mentally represent collaboration?: Action-learning and communication in a partnered task**

Manuscript: 3176724

Authors: Elizabeth Warren, Emma Suvi McEwen , Josep Call

Introduction

Page 8

Line 139 Here the authors use the term 'reestablish', rather than the term 're-establish' but later use the hyphen for other terms (e.g. 're-initiate', line 143). A spelling rule should be chosen and applied consistently throughout.

Materials and methods

Page 13

Lines 227-230 The description here of the structure of the apparatus could be made clearer. Perhaps it could be described as having a ridge on one terminal/short end and a depression on the other terminal/short side - just to clarify that the ridge and groove are not located along the longitudinal sides of the apparatus (which could be how it might be interpreted from the text as it is presented here).

Pages 14-15

Lines 258-274 How were tests carried out in relation to general husbandry? Were tests conducted during a specific time window during each day? How long after/prior to feeding were tests conducted? These details should be made explicit given that the study involves cognitive testing and food rewards. It is possible that arousal due to recent/upcoming events may influence an individual's ability to focus and complete the task. Similarly, satiety or hunger will influence the level of motivation of individuals and thereby their performance in the task.

Page 18

Line 347 The word 'flip' does not seem like an intuitive descriptor for the outcome described. ‘Flip’ implies a rotation of 180° or more along a given axis. Perhaps 'tip' would be a better term to use here and throughout?

Discussion

Page 28

Lines 523-525 The grammar in this sentence seems off. Perhaps consider rewording.

Page 30

Lines 562-562 It is also possible that individuals vary in their capacity for causal reasoning and thus their ability to understand the mechanism of the apparatus. It is a pity that the sample size in this study is limited - the possible roles of age and sex cannot reasonably be explored as a result but these potentially may be influential. It may be worthwhile briefly discussing these aspects as well.
